# Association between CYP19 gene SNP rs2414096 Polymorphism and polycystic ovary syndrome in Chinese women

**DOI:** 10.1186/1471-2350-10-139

**Published:** 2009-12-16

**Authors:** Jia-Li Jin, Jing Sun, Hui-Juan Ge, Yun-Xia Cao, Xiao-Ke Wu, Feng-Jing Liang, Hai-Xiang Sun, Lu Ke, Long Yi, Zhi-Wei Wu, Yong Wang

**Affiliations:** 1 Jiangsu Key Laboratory of Molecular Medicine & The reproductive medicine Center of Drum Tower Hospital, Medical School of Nanjing University, Nanjing 210093, China; 2 Department of Obstetrics and Gynecology, Anhui Medical University, Hefei 230022, China; 3 Department of Obstetrics and Gynecology, the First Affiliated Hospital, Heilongjiang University of Chinese Medicine, Harbin 150040, China

## Abstract

**Background:**

Several studies have reported the association of the SNP rs2414096 in the CYP19 gene with hyperandrogenism, which is one of the clinical manifestations of polycystic ovary syndrome (PCOS). These studies suggest that SNP rs2414096 may be involved in the etiopathogenisis of PCOS. To investigate whetherthe CYP19 gene SNP rs2414096 polymorphism is associated with the susceptibility to PCOS, we designed a case-controlled association study including 684 individuals.

**Methods:**

A case-controlled association study including 684 individuals (386 PCOS patients and 298 controls) was performed to assess the association of SNP rs2414096 with PCOS. Genotyping of SNP rs2414096 was conducted by the polymerase chain reaction-restriction fragment length polymorphism (PCR-RFLP) method that was performed on genomic DNA isolated from blood leucocytes. Results were analyzed in respect to clinical test results.

**Results:**

The genotypic distributions of rs2414096 (GG, AG, AA) in the CYP19 gene (GG, AG, AA) in women with PCOS (0.363, 0.474, 0.163, respectively) were significantly different from that in controls (0.242, 0.500, 0.258, respectively) (*P *= 0.001). E2/T was different between the AA and GG genotypes. Age at menarche (AAM) and FSH were also significantly different among the GG, AG, and AA genotypes in women with PCOS (P = 0.0391 and 0.0118, respectively). No differences were observed in body mass index (BMI) and other serum hormone concentrations among the three genotypes, either in the PCOS patients or controls.

**Conclusions:**

Our data suggest that SNP rs2414096 in the CYP19 gene is associated with susceptibility to PCOS.

## Background

Polycystic ovary syndrome (PCOS) is a heterogeneous disease affecting 7.4% of fertile women in China [1]. This syndrome is characterized by chronic anovulation, clinical and/or biochemical hyperandrogenism, and polycystic ovary, commonly leading to infertility [2-5]. Recently, many findings have enlightened us with information regarding the genetic background of PCOS, suggesting that genetic factors are involved in the etiology of the disorder [6].

Ovarian androgen overproduction is the key physiopathologic feature of PCOS [7]. A number of genes encoding major enzymes of the androgen metabolic pathways, such as HSD17B6, CYP19, CYP11A1, CYP17, and INSR, have been examined and associations reported [3,8-11] , although support for these associations has not been unanimous.

The CYP19 gene is located on the long arm of chromosome 15 at position 15q21.1 [12]. It encodes aromatase (P450arom), a key steroidogenic enzyme that catalyzes the final step of estrogen biosynthesis by converting testosterone and androstenedione to estradiol and estrone separately [13]. It is reported that several single nucleotide polymorphisms (SNPs) of the CYP19 gene were associated with variation in serum androgen concentrations among women, both within and between racial/ethnic groups. Several studies have reported the association of the SNP rs2414096 in the CYP19 gene with hyperandrogenism [3,14]. These studies suggest that altered regulation of this enzyme may be involved in PCOS.

In view of the strong evidence implicating the importance of CYP19 SNP rs2414096 in androgen metabolic pathways, we investigated the impact of such gene polymorphisms on susceptibility of developing PCOS, especially characterized by clinical or biochemical hyperandrogenism in Chinese subjects.

## Methods

### Subjects

A total of 684 individuals, including 386 PCOS patients and 298 non-PCOS control women (some of them with one child or more) with normal menstrual cycles (< 32 days) and without obesity, hirsutism, cystic acne, overmuch sebum, and insulin resistance, were studied. All the participants recruited for our study were of Chinese Han origin, a predominant Chinese ethnic population. The study was approved by Medical School of Nanjing University, and informed consent was obtained from each study participant.

### PCOS diagnostic criteria and hormone measurements

Patients with PCOS were diagnosed by the 2003 Rotterdam Criteria [15] (The Rotterdam ESHRE/ASRM-Sponsored PCOS Consensus Workshop Group, 2004). The Rotterdam Criteria requires at least two of the following indicators for diagnosis of PCOS: clinical or biochemical signs of hyperandrogenism, oligomenorrhea or amenorrhea, and presence of polycystic ovarian (PCO) morphology on ultrasound, with the exclusion of other causes of hyperandrogenism such as hyperprolactinemia, androgen-secreting tumors, Cushing's syndrome and nonclassic congenital adrenal hyperplasia.

We obtained the participants' age at menarche (AAM) through inquiry and calculated the body mass index (BMI = body weight in kilograms divided by square of height in meters) to assess obesity. Peripheral blood was obtained by a single venipuncture during the 3rd to the 5th day of the menstrual cycle for those who had menstruation and at any time for those who had amenorrhea. All peripheral blood samples were obtained between 8 AM and 9 AM after a 12-hour overnight fast. None of the study participants had been taking hormonal medications, e.g. contraceptive pills, for the previous three months before the hormone measurement. Blood samples were immediately centrifuged and then serum was separated and frozen at -80°C until assayed. Levels of total testosterone (T), follicle-stimulating hormone (FSH), luteinizing hormone (LH), and estradiol (E2) in the sera were measured by RIA (Beijing North Institute of Biological Technology of China and the CIS Company of France). The E2/T ratio was used as an index of the aromatase activity. All assays had intra- and inter-assay coefficients of variation less than 10%.

### Polymorphism genotyping analysis

Genomic DNA was isolated from human leukocytes by using Chelex^®^-100 as a medium (Promega, Madison, WI, USA). Genotyping of the rs2414096 polymorphism of the CYP19 gene was performed with the polymerase chain reaction-restriction fragment length polymorphism (PCR-RFLP) method. The sequences of the primers were 5'-TCT GGA AAC TTT TGG TTT GAG TG-3' (forward primer) and 5'-GAT TTA GCT TAA GAG CCT TTT CTT ACA-3' (reverse primer). PCR amplification was carried out in a total volume of 25 μL containing 50 ng of genomic DNA, 6.25 pmol of each primer, 2.5 μL STR (short tandem repeat), 10×buffer (STR 10×buffer, Promega, Madison, WI, USA) and 1.5 U of Taq DNA polymerase (Promega, Madison, WI, USA). The PCR was performed in a PTC-100 (MJ Research™, Incorporated) thermocycler as follows: 30 cycles consisting of 1 minute of denaturation at 94°C, 1 minute of annealing at 60°C, and 1 minute of extension at 72°C. An initial denaturation step of 5 minutes at 94°C and a final extension of 10 minutes at 72°C were used. The PCR products of 189-bp were then digested with HSP92 II at 37°C overnight. A single 189 bp band corresponds to the wild-type G homozygote; bands of 189, 161, and 28 bp stand for the AG heterozygotes; and 161 and 28 bp for the A homozygote. The DNA fragments were separated by electrophoresis on a 2% agarose gel, and visualised by staining with ethidium bromide.

### Statistical analysis

Fisher's Exact Test was used to compare the CYP19 gene genotype distributions in the case-control study. The analysis was performed using the SAS system software (SAS Institute Inc., Cary, NC 27513-2414 USA). The results of serum hormone levels are reported as means ± SD. Clinical variables such as age and BMI, and AAM were compared using one-way analysis of variance (ANOVA). Differences in serum hormone levels among different genotypic individuals were assessed using analysis of covariance (ANCOVA) to correct for age and BMI. P < 0.05 was considered statistically significant. Hardy-Weinberg distribution of genotypes in the PCOS and control groups was assessed.

## Results

In 684 study subjects (Table 1), the CYP19 rs2414096 genotypic distributions were 0.310 for GG, 0.485 for AG and 0.205 for AA. Genotypic distributions (GG, AG and AA) in women with PCOS (0.363, 0.474, 0.163, respectively) were significantly different from the controls (0.242, 0.500, 0.258, respectively) (p = 0.001) (Table 2). All the genotypic distributions were in Hardy-Weinberg equilibrium.

**Table 1:** The overall characteristics of PCOS patients and controls

_	N	Age* (years)	AAM (years)	BMI* (kg/m^2^)	FSH (IU/L)	LH* (IU/L)	LH/FSH*	T* (nMol/L)	E2* (pMol/L)
PCOS	386	26.6 ± 4.2	14.4 ± 1.6	22.7 ± 3.5	7.3 ± 3.9	15.1 ± 6.9	2.4 ± 1.2	2.9 ± 1.5	223.6 ± 136.2
CONTROL	298	31.5 ± 4.43	14.4 ± 1.3	21.4 ± 2.3	7.1 ± 2.1	4.5 ± 2.0	0.6 ± 0.3	1.1 ± 0.7	166.1 ± 142.5

Note: *P < 0.05 vs control.

**Table 2:** The frequency distribution of CYP19 RS2414096 in women with PCOS and controls

RS2414096	Alleles N (%)		P	Genotypes N (%)			P
				
	A	G		AA	AG	GG	
Control	303(50.8)	293(49.2)	0.001	77(0.258)	149(0.5)	72(0.242)	0.001
PCOS	309(40.0)	463(60.0)		63(0.163)	183(0.474)	140(0.363)	

E2/T was different between the AA and GG genotypes (Tukey-test). Age at menarche and level of FSH were also significantly different among GG, AG, and AA genotypes in women with PCOS (P = 0.0391 and 0.0118, respectively). There were no significant differences in BMI and levels of other serum hormones, such as total T, LH, and E2 (Table 3), among the three genotypes of CYP19 rs2414096 both in the patients with PCOS and the controls.

**Table 3:** Anthropometric characteristics and serum hormone concentrations in different genotypic women with PCOS and controls

	PCOS				CONTROL			
				
Genetype	AA	AG	GG	P-value	AA	AG	GG	P-value
Age (years)	27.08 ± 4.02	26.64 ± 4.6	26.53 ± 3.75	0.8061	30.65 ± 4.98	31.48 ± 4.42	32.57 ± 4.14	0.292
Menarche (years)	13.88 ± 1.48	14.85 ± 1.66	14.33 ± 1.68	0.0391	14.22 ± 1.01	14.52 ± 1.51	14.54 ± 1.48	0.6225
BMI (kg/m^2^)	23.67 ± 3.16	22.79 ± 3.80	22.31 ± 3.33	0.1619	20.88 ± 2.32	21.5 ± 2.26	21.78 ± 2.52	0.3492
FSH(IU/L)	6.35 ± 2.66	6.83 ± 3.98	8.34 ± 4.23	0.0118	6.46 ± 1.95	7.43 ± 2.48	6.97 ± 1.73	0.1674
LH(IU/L)	14.72 ± 8.05	18.54 ± 21.33	20.23 ± 16.68	0.3307	4.19 ± 2.7	4.73 ± 1.76	4.52 ± 2.03	0.5572
LH/FSH	2.55 ± 1.56	2.68 ± 1.47	2.49 ± 1.05	0.6293	0.65 ± 0.37	0.67 ± 0.28	0.68 ± 0.41	0.9569
T (nMol/L)	2.68 ± 1.55	2.87 ± 1.53	3.19 ± 1.43	0.2241	0.89 ± 0.62	1.04 ± 0.58	1.16 ± 0.73	0.3131
E2 (pMol/L)	204.57 ± 128.63	230.9 ± 145.56	221.77 ± 127.17	0.6461	152.72 ± 129.43	171.22 ± 122.27	170.02 ± 186.88	0.8525
E2/T (ln)	4.15 ± 0.85	4.10 ± 0.61	3.81 ± 0.72	0.0444	5.02 ± 1.24	4.95 ± 0.99	4.93 ± 0.92	0.9431

Note: value = Means ± SD

## Discussion

There were marked differences in allele frequencies for the SNP rs2414096 in CYP19 gene in this case-control study. The frequency of the A allele in the PCOS patients was lower than that in the controls. The significant difference in allele distribution probably indicates that the SNP of rs2414096 in CYP19 gene is associated with the aromatase activity in PCOS women. The enzyme aromatase, the single gene product of *CYP 19*, mediates the conversion of the androgens testosterone and androstenedione to the estrogens estradiol (E2) and estrone, respectively, in gonadal and extragonadal tissues.

CYP 19 rs2414096 is located in an intron and therefore does not affect the protein sequence of aromatase. However, mutations in introns sometimes can be associated with regulatory sequences. The E2/T ratio provides important information about aromatase activity because conversion of androgens to estrogens is mediated by CYP19, which suggeststhat E2/T ratio may be a direct marker of aromatase activity. Our study demonstrates that the rs2414096 A allele may be associated with activity of the aromatase and further affect the conversion of androgens to estrogens. The E2/T ratio of the AA genotype in PCOS was significantly higher than that of the other two genotypes and this suggests that aromatase activity was augmented in the AA genotype. Reduced aromatase activity may lead to ovarian hyperandrogenism and the development of PCOS, which can be deduced from the facts that a higher frequency of PCOS is observed in people with aromatase deficiency caused by rare loss-of-function mutations [16-18] and antral follicles taken from PCOS women exhibits no aromatase activity [19]. The augmented activity of the aromatase in the AA genotype may protect the ovary from the development of hyperandrogenism in PCOS patients. Our results were in contrary to that reported by C.J.Petry et.al, [3], who found that the 'A' allele, which was more prevalent in precocious pubarche (PP) girls is associated with increased testosterone concentrations in both the Barcelona PP case-control study and the Oxford population study. The fact that our results were different from that of Petry et al's may lie in these points: 1) The selection criteria for the subjects is different. Petry et al chose the girls with PP (Age at assessment = 9.8-10.9) as participants, while our subjects were women with PCOS (Age at assessment = 21-33). Adolescent girls are subject to physical and psychological changes dramatically, and their endocrine levels fluctuate more to internal and external environmental factors. 2) The frequency distribution of alleles A/G is different between Asians and Europeans. 3) In Petry et al's experiment, the SNP 50 genotypes from girls with PP were not in Hardy-Weinberg equilibrium (P < 0.05). Their sample size is small and it may not be representative of the overall ensemble, thus affecting the statistical accuracy.

FSH can induce aromatase activity, which is positively correlated to the E2 level. Thus, a reduced E2 level can stimulate the production of FSH by negative feedback. This may account for our observation that the concentration of FSH in the GG genotype, which demonstrated lower aromatase activity, was higher compared with the other two genotypes (table 3)

In our studies we also found that the AA genotype of rs2414096 was associated with age at menarche in PCOS women. C.J.Petry et al [3] also found that the distributions of the aromatase SNP-50 (rs2414096) genotype were significantly different between PP girls and controls in Spanish subjects. Taken together, these observations suggest that the AA genotype of the SNP of rs2414096 in CYP19 gene could affect the function of the aromatase and contribute to the development of PCOS in the adolescent girls. This AA genotype is associated with increased activity of aromatase and high levels of E2, resulting in advanced puberty.

This study suggests that the SNP of rs2414096 in CYP19 gene is positively associated with PCOS. However, whether this SNP is a direct causal factor or it is a parallel phenomenon due to linkage disequilibrium (LD) with other genetic mutations is not known. The exact morbific site still needs to be investigated and located. Several other studies show that variation in the *CYP19 *gene is unlikely to be responsible for PCOS by investigating both a tetranucleotide repeat (TTTA) polymorphism in intron 4 (the different numbers of tetranucleotide TTTA repeats in intron 4 are associated with increased risk for breast cancer) and the promoter of the *CYP19 *gene [12,20,21]. However, since only some SNPs have been described to be associated with PCOS, we cannot predict whether the other sites are also associated with the etiopathogenesis of PCOS. It's necessary to assess the polypmorphisms of the entire CYP19 using the method of Haplotype-tags before we can obtain any conclusion.

We failed to demonstrate the differences of the T levels among the three genotypes of rs2414096. The etiopathogenesis of PCOS starts in adolescence which is the key stage of the development of PCOS. The aromatase is closely associated with the concentrations of androgen and estrogen and it is very important for the development of ovary during the adolescence. C.J.Petry at al. [3] found that the aromatase (CYP 19) gene SNP_50 (rs2414096) is associated with features of hyperandrogenism in two populations of young women. But for adults, the concentrations of androgen and estrogen may be regulated by many other factors including environmental factors and life styles, so it may not be closely associated with a single factor of the SNP of rs2414096 in the CYP19 gene.

In conclusion, to our knowledge, this is the first report demonstrating that the SNP rs2414096 in the CYP19 gene is one of the key factors responsible for the etiopathogenisis of PCOS, especially in adolescence. It may be associated with the activity of the aromatase. The A allele can stimulate aromatase activity and catalyze the conversion of testosterone and androstenedione to estradiol and estrone. This effect is more pronounced with higher concentrations of androgen, which can lead to menarche at an earlier age.

## Conclusions

This study suggests that polymorphisms of rs 2414096 in *CYP19 *are associated with the pathogenesis of PCOS.

## Authors' contributions

JJ, JS, HJG, FL, LK carried out DNA extraction and the molecular genetic studies. JJ, JS, HJG, ZWW, LY, YW performed the statistical analysis and drafted the manuscript. YC, XW, HS participated in sample collection. L Y participated in the design of the study and helped to carry out the molecular genetic studies. YW conceived the study, and participated in its design and coordination and helped to draft the manuscript. All authors read and approved the final manuscript.

## Acknowledgements

We are extremely grateful to all the women who agreed to participate in our study. This study was supported by the National Natural Science Foundation of China (30672228) and the Natural Basic Research Program of China (973 program 2010CB945103).

## Pre-publication history

The pre-publication history for this paper can be accessed here:

http://www.biomedcentral.com/1471-2350/10/139/prepub
